# Labour outcomes in caseload midwifery and standard care: a register-based cohort study

**DOI:** 10.1186/s12884-018-2090-9

**Published:** 2018-12-06

**Authors:** Ingrid Jepsen, Svend Juul, Maralyn Jean Foureur, Erik Elgaard Sørensen, Ellen Aagaard Nohr

**Affiliations:** 1 University College of Northern Denmark, Selma Lagerløfs Vej 2, 9220 Aalborg Øst, Denmark; 2 Clinical Nursing Research, Aalborg University Hospital, Sdr. Skovvej 15, 9000 Aalborg, Denmark; 3 Department of Clinical Medicine, Aalborg University, Sdr. Skovvej 15, 9000 Aalborg, Denmark; 4 Section for Epidemiology, Department of Public Health, Aarhus University, Bartholins Alle 2, 8000 Aarhus C, Denmark; 5 Research unit for Gynaecology and Obstetrics, Institute of Clinical Research, University of Southern Denmark, Sdr Boulevard 29, 5000 Odense C, Denmark; 6 Centre for Midwifery, Child and Family Health, Faculty of Health, University of Technology Sydney, Sydney, Australia; 7 Centre of Women’s, Family and Child Health, University of South-Eastern Norway, Kongsberg, Norway

**Keywords:** Caseload midwifery, Labour outcome, Cohort study

## Abstract

**Background:**

Research on caseload midwifery in a Danish setting is missing. This cohort study aimed to compare labour outcomes in caseload midwifery and standard midwifery care.

**Methods:**

A historical register-based cohort study was carried out using routinely collected data about all singleton births 2013–2016 in two maternity units in the North Denmark Region. In this region, women are geographically allocated to caseload midwifery or standard care, as caseload midwifery is only available in some towns in the peripheral part of the uptake areas of the maternity units, and it is the only model of care offered here. Labour outcomes of 2679 all-risk women in caseload midwifery were compared with those of 10,436 all-risk women in standard midwifery care using multivariate linear and logistic regression analyses.

**Results:**

Compared to women in standard care, augmentation was more frequent in caseload women (adjusted odds ratio (aOR) 1.20; 95% CI 1.06–1.35) as was labour duration of less than 10 h (aOR 1.26; 95% CI 1.13–1.42). More emergency caesarean sections were observed in caseload women (aOR 1.17; 95% CI 1.03–1.34), but this might partly be explained by longer distance to the maternity unit in caseload women. When caseload women were compared to women in standard care with a similar long distance to the hospital, no difference in emergency caesarean sections was observed (aOR 1.04; 95% CI 0.84–1.28).

Compared to standard care, infants of caseload women more often had Apgar ≤7 after 5 min. (aOR 1.57; 95% CI 1.11–2.23) and this difference remained when caseload women were compared to women with similar distance to the hospital.

For elective caesarean sections, preterm birth, induction of labour, dilatation of cervix on admission, amniotomy, epidural analgesia, and instrumental deliveries, we did not obseve any differences between the two groups. After birth, caseload women more often experienced no laceration (aOR 1.17; 95% CI 1.06–1.29).

**Conclusions:**

For most labour outcomes, there were no differences across the two models of midwifery-led care but unexpectedly, we observed slightly more augmentation and adverse neonatal outcomes in caseload midwifery. These findings should be interpreted in the context of the overall low intervention and complication rates in this Danish setting and in the context of research that supports the benefits of caseload midwifery. Although the observational design of the study allows only cautious conclusions, this study highlights the importance of monitoring and evaluating new practices contextually.

**Electronic supplementary material:**

The online version of this article (10.1186/s12884-018-2090-9) contains supplementary material, which is available to authorized users.

## Introduction

Internationally, there has been a growing focus on how to improve care for pregnant and labouring women by providing continuity of care through caseload midwifery [1–3]. Caseload midwifery aims to ensure that childbearing women receive their ante-, intra- and postnatal care from a known midwife or her colleague(s) [3, 4]. Caseload midwifery is internationally well known as many countries have explored and implemented variations of this model of care [3, 5].

In Denmark, midwives were authorised 300 years ago and midwife-led practice has been the standard model of care for all childbearing women since then [6]. In accordance with the International Definition of the Midwife [7], Danish midwives are authorised to be in charge of managing uncomplicated childbirth. If complications arise, midwives will refer to obstetricians, but will continue to provide care for these women throughout labour [8]. In 1974, childbirth was hospitalized in Denmark [6] and after that, antenatal care was centralised in community centers. The midwives working in these centers are also rostered to care for women during labour through working in shifts at the hospital [9]. This means that the care is still midwife-led, but in most situations, although continuity of care during labour and birth is prioritized, women will not be attended by a known midwife during labour.

In the Danish model of caseload midwifery, continuity of care in both pregnancy and childbirth is the focus [10]. Midwives typically work in pairs or small groups succeeding each other with days on call followed by days of leisure time. In standard maternity care, most full time midwives are rostered to work 37 h a week. They follow women during their pregnancy but not through labour [11]. All pregnant women are offered 3 antenatal consultations with a general practitioner, 4–7 midwifery consultations, typically with the same midwife, and are referred to an obstetrician if complications arise during pregnancy or in labour. In contrast to other countries, health-care nurses provide postnatal care, and midwives have only one contact with the family after birth.

Caseload midwifery is increasing in Denmark, and 61% of public maternity hospitals have implemented some kind of caseload practice [11]. The growing interest in caseload midwifery in Denmark builds on the results from international research showing lower intervention and complication rates during labour and birth when midwife-led continuity of care models are used [1–3, 12–14]. In a recent Danish evaluation report on caseload midwifery from the Central Denmark Region, the generalisability of the results from international studies was questioned [15]. While standard Danish care in pregnancy and labour is led by midwives, many other countries have standard care led by a mixture of health professionals (midwives, general practitioners, and obstetricians), whereas midwife-led care is reserved for midwives working in caseloads [2, 16]. The report concluded that the outcomes of Danish standard care and caseload midwifery might be more similar than seen in other countries because differences between the models of care are smaller [15].

It is important to investigate if or how caseload midwifery is associated with the intervention and complication rates in Danish labour wards. In The North Denmark Region, women are geographically allocated to caseload midwifery or standard care. Caseload midwifery is only available in some areas in the peripheral part of the maternity units’ uptake areas, and in these areas it is the only model of care offered. Using data from two maternity units in this region, this study aimed to compare labour and birth outcomes between caseload midwifery and standard care.

The hypothesis was that compared to international findings this study might find none or only small differences in outcomes in favor of caseload midwifery because both models of care are midwife-led.

## Methods

### Design

A register-based historical cohort study was performed in the North Denmark Region including data from a tertiary maternity unit with approximately 3200 births a year (maternity unit A) and a secondary unit with around 1300 births a year (maternity unit B).

During a 3-year inclusion period from 1 January 2013 to 31 December 2015, data on all women giving birth were retrieved from the local electronic obstetric database of the North Denmark Region. After excluding women with multiple pregnancies (*n* = 253), 13,115 women with singleton all-risk pregnancies remained in the study population. The local obstetric database provides data to the Danish Medical Birth Register, but contains more detailed information than the national register.

### Description of caseload midwifery and standard care

During the study period, full time caseload midwives in the region were expected to attend 60 all-risk pregnant women a year, to conduct pregnancy consultations in small antenatal clinics, and to attend their caseload during labour and birth, mainly in hospitals. The number of babies born at home in planned home births during the three years was 259 (2%) .

Full time midwives working in standard care had 37 h rostered work at the maternity unit per week and were expected to provide care during labour and birth for on average around 75 all-risk women per year. Typically, their weekly schedule included one day in the antenatal clinic where they provided continuity of care during pregnancy for a group of pregnant women, but rarely they would be able to attend them during labour and birth. Both caseload and standard care midwives managed the care for women with uncomplicated pregnancies and births and for women with complications of pregnancy or labour in cooperation with obstetricians [11].

In maternity unit A and B, respectively 15.5 and 32.9% of midwives worked in caseloads (in total 16.9 out of 72.3 full time equivalents in these two maternity units). There were 8 caseload groups at the two maternity units. In 7 caseloads, the midwives worked in pairs succeeding each other with one week on call and one week of leisure time while one caseload consisted of three midwives who worked a similar rotation but had fewer continuous days on call. Caseload and standard care midwives collaborated: after 12–16 h at work as a caseload midwife, midwives from standard care took over until the caseload midwife had rested; the caseload midwives did not back up each other. In a peak situation at the hospital where all available midwives were at work, the caseload midwives on call could be required to work as standard care midwives. In Maternity unit A, caseload midwives were on average required to do standard care 12 times per midwife per year in 2015. In Maternity unit B, this number was 5 times per midwife.

The option of caseload midwifery was reserved for pregnant women living in the peripheral part of the uptake areas of the maternity units. In some peripheral towns, caseload midwifery was the only available model providing maternity care. Thus, women did not actively choose caseload midwifery; rather they were allocated to a caseload because of their geographical location.

### Outcome measures and confounders

The exposure in this study was caseload midwifery. When a caseload midwife was registered in the obstetric database as a woman’s midwife during antenatal care (primary midwife hereafter), the woman was defined as belonging to caseload midwifery. The codes of the midwives were also attached to procedures and interventions, and this allowed an investigation of the degree of continuity of care.

Outcome variables were categorized according to the International Classification of Diseases, tenth revision (ICD-10) [17]. Outcome variables included elective (before labour) and emergency (during labour) caesarean section, induction, augmentation with syntocinon, amniotomy, epidural anaesthesia for pain relief in labour (not for anaesthesia during CS) and instrumental delivery. Information on dilatation on admission was included in the database in November 2014 and was not available for the full study period. We also investigated the differences in lacerations of 1–2 degrees, lacerations of 3–4 degrees, early discharge, time in hospital after birth, length of labour < 10 h, and the mean length of labour. To address neonatal outcomes, we defined ‘low Apgar score’ as Apgar score ≤ 7 after one and five minutes which is in accordance with the definition in other studies [1, 3, 12]. We also addressed umbilical pH immediately after birth and transfer to the Neonatal Care Unit (NCU).

We counted the number of midwives attending each labouring woman and the number of procedures carried out by her primary midwife to examine the degree of continuity of care. To measure how frequently a primary midwife was present when a woman gave birth, we defined the variable “known midwife at birth” where at least one of three criteria had to be fulfilled: A primary midwife 1) signed the procedures within 30 min before and after labour, 2) established skin to skin contact, or 3) reported “continuous presence of a midwife” in the medical record.

Information about the following potential confounders, selected a priori, was retrieved from the database: maternal age, parity (nulliparous vs multiparous), maternal pre-pregnancy body mass index (BMI) (derived from pre-pregnancy weight and height), smoking habits (non-smoker, smoker, stopped during pregnancy), need for an interpreter (yes/no), maternity unit (A or B), infant birthweight (< 3000 g, 3000–3999 g, ≥4000 g), and infant’s birth year (2013, 2014, 2015). Because of the geographical allocation of caseload midwifery, social status might act as a confounder, and therefore permission was obtained to add “Mothers years in school” and “Level of education” to the database in November 2014. These variables were grouped as more or less than 9 years in school and more or less than three years of professional education.

Based on the database, we also constructed the variable “pre-pregnancy risks” including previous intrauterine growth restriction, caesarean section, and preterm birth, and the variable “risk factors/complications in the present pregnancy” including alcohol or drug abuse, in vitro fertilisation, preeclampsia, hypertension, diabetes, premature contractions < 37 weeks gestation, vaginal bleeding < 37 weeks gestation, placental and uterine abnormalities, congenital malformations, and blood type incompatabilities (rhesus, ABO, platelets, hydrops foetalis and other kinds of blood type incompatability).

### Statistics

Demographic characteristics and outcomes were compared across the two models of care using chi-squared test for proportions, Student’s t-test for normally distributed data, and Wilcoxon rank-sum test for non-normally distributed data. The mean number of midwives per labour and the mean number of procedures carried out by a primary midwife were compared using Student’s t-test. The chance of being attended by a primary midwife during birth was calculated by bivariate analyses of the variable “known midwife at birth” which was defined in the previous section.

To compare interventions and labour outcomes in caseload midwifery and standard care, we used logistic or linear regression, depending on whether the outcome variable was dichotomous or continuous. In the adjusted analyses, we included as continuous variables maternal age and BMI and as categorical variables parity, birthweight, smoking habits, need for an interpreter, maternity unit, birth year, pre-pregnancy risks, and risk factors in the present pregnancy. The inclusion of social variables in the register during the study period allowed us to control for social status only in 14 months of the 3-year study period. The main adjusted model was repeated for this specific time period and compared to a model where years in school and level of education were added to the model to examine potential confounding by these factors.

We performed several supplementary analyses. Because only women in smaller, distant towns/areas were allocated to caseload midwifery and because travel time to hospital has been associated with adverse neonatal outcomes [18], we attempted to examine the impact of transport time. Thus, we compared caseload women to women in standard care attending antenatal clinics with a similar distance to the hospital. Moreover, we restricted the analyses to women at “low risk” and also stratified the analyses by parity to provide estimates for both primiparous women and multiparous women. Finally, we stratified the analyses by maternity unit.

Due to the geographical allocation of caseload midwifery, women from two centers for refugees were included in caseload midwifery. We therefore repeated the analyses after excluding women needing an interpreter (5.0% in caseload midwifery, and 2.4% in standard care). Finally, we repeated the analyses after excluding all homebirths.

All estimates were presented with 95% confidence intervals. As the residuals from the linear regression analyses were judged to be not normally distributed, we used bootstrap analysis to estimate confidence intervals. A correction for within-cluster correlation (robust standard errors) was applied in all reported analyses, because some women had more than one birth during the study period (*n* = 1693). All statistical analyses were carried out using Stata 13 [19].

## Results

Of all 13,115 births, 20.4% (2679) were allocated to caseload midwifery. Baseline characteristics of the study population are presented in Table 1. Across the study period, the number of caseload women decreased somewhat. Most births (71.8%) happened in Birth Unit A.

**Table 1:** Baseline characteristics according to caseload midwifery and standard care

	Total *n* = 13,115	Caseload Midwifery *n* = 2679			Standard Care *n* = 10,436		
	%	%		n	%		n
Singleton births (*n* = 13,115)		100		2679	100		10,436
Births per year							
2013	32.1	36.5		978	31.0		3230
2014	33.2	33,4		896	33.1		3456
2015	34.7	30.0		805	35.9		3750
Births per maternity unit:							
Maternity unit A	71.8	45.4		1217	76.3		7958
Maternity unit B	28.2	54.6		1462	23.7		2478
Years school = < 9 years ^a^	25.4	30.7		263	24.2		958
Professional education = < 3 years ^a^	34.4	40.7		349	33.0		1307
Married	42.5	45.5		1219	41.7		4350
Living alone	5.8	5.8		155	5.8		604
Need of interpreter	2.9	5.0		135	2.4		247
Smoking in pregnancy							
no	89.5	88.0		2357	89.9		9386
yes	9.0	10.3		276	8.7		905
ceased during pregnancy	1.5	1.7		46	1.4		145
Complicated pregnancy^b^	22.7	23.1		618	22.7		2365
Pre-pregnancy risks	26.7	26.7		714	26.8		2793
Parity 0	46.8	41.2		1104	48.2		5027
Parity 1	36.5	36.3		973	36.6		3814
Parity 2	12.2	15.7		420	11.3		1175
Parity 3	3.2	4.7		125	2.9		300
Parity > = 4	1.3	2.1		57	1.1		116
	Total mean	Mean	SD	n	Mean	SD	n
Visits General Practitioner	2.7	2.7	0.9	2610	2.7	0.8	10,107
Visits Midwife	5.0	5.1	1.6	2610	5.0	1.6	10,111
Visits Obstetrician	3.7	3.7	2.3	2608	3.8	2.3	10,110
Mothers age (years)	30.0	29.8	5.0	2679	30.0	5.0	10,436
Mothers height (cm)	167	167	6.6	2678	167	6.6	10,433
Mother weight at hosp.(kg)	84.2	85.9	17	1223	83.8	15.8	6300
Prepregnancy BMI	24.9	25.7	5.5	2678	24.7	5.2	10,430
Gestational age(days)	278	278	13.4	2679	278	14.6	10,432
Infant length (cm)	51.6	51.7	2.9	2654	51.6	3.0	10,393
Infant weight (g)	3500	3521	580	2673	3495	592	10,427

^a^Data only available from 1 November 2014 to 31 January 2015 (*n* = 4815)

^b^complicated pregnancy included: malformations; alcohol or drug abuse; IVF; primiparous< 20; preeclampsia; hypertension; diabetes; premature contractions < 37 weeks of gestation; vaginal bleeding < 37 weeks of gestation; placental abnormalities; uterine abnormalities, and blood type incompatibilities (Rh, ABO, platelets, hydrops foetalis, and other kinds of blood type incompatibilities)

There were no differences according to mothers’ age and height and the number of women living alone, although caseload women were more likely to be legally married. Women in standard care tended to attend more years in school, have a higher level of education, and lower weight and pre-pregnancy BMI than women in caseload midwifery. Caseload women were less likely to be primiparous, more likely to smoke during pregnancy, and more often needed an interpreter. There were no differences in types and numbers of antenatal visits. Obstetric pre-pregnancy risks and complications during pregnancy were similar as were mean gestational age at birth and infant-length. On average, infants born in the caseload model were 26 g heavier than standard care infants.

**Fig. 1:**
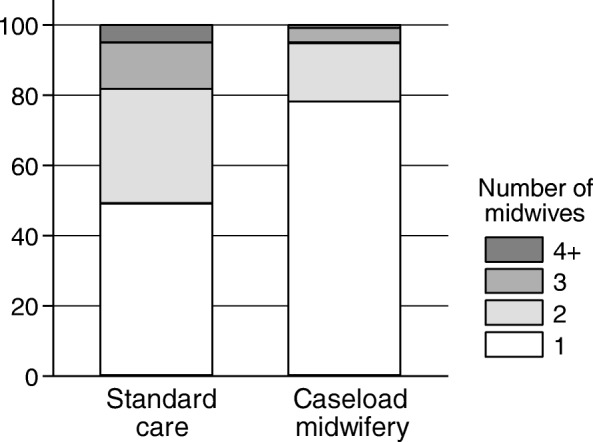
Number of different midwives during labour in standard care and caseload midwifery

As shown in Fig. 1, 78% of the caseload women had only one midwife present throughout labour compared to 49% in standard care. One or two midwives attending the birth occurred in 95% of caseload births, and in 82% of standard care births. The mean number of midwives during labour was 1.3 (standard deviation (SD) 0.6) in caseload midwifery and 1.8 (SD 0.9) in standard care (*p* < 0.0001).

Among caseload women, 70% of all procedures were undertaken by a primary midwife compared to only 5% in standard care (*p* < 0.0001). Also, 70% of caseload women and 5% of standard care women were attended by a primary midwife when giving birth (calculations not shown).

In Table 2, comparisons of birth outcomes are shown. Although crude estimates indicated that women in caseloads had slightly more elective Cesarean sections and fewer epidurals and instrumental deliveries, these differences were not present after adjustment. Preterm birth, induction of labour, dilatation of cervix on admission, and amniotomy, were similar in the two groups. While crude frequencies of augmentation (use of syntocinon drip) were similar, the adjusted odds for augmentation were higher in caseload midwifery (adjusted OR 1.20 (95% CI 1.06–1.35)) with parity being the most influential confounder. Also, adjusted odds for emergency caesarean sections were higher in caseload midwifery (adjusted OR 1.17 (95% CI 1.03–1.34)). The adjusted odds for having a labour lasting less than 10 h were higher in the caseload group (adjusted OR 1.26 (95% CI 1.13–1.42)) (Table 2), and the duration of labour was on average 28 min shorter than in the standard care group (Table 3). Among caseload women, adjusted odds for having no laceration after birth were increased (adjusted OR 1.17 (95% CI 1.06–1.29)) (Table 2), mainly explained by lower odds for 1st or 2nd degree lacerations (adjusted OR 0.86 (95% CI 0.77–0.95)), while there was no difference in 3rd or 4th degree lacerations across the two groups. Although crude estimates suggested that caseload women more often experienced early discharge (Table 2) and had a shorter hospital stay after birth (Table 3), no differences in early discharge were observed after adjustment.

**Table 2:** Labour outcomes in caseload midwifery and standard care

	Caseload Midwifery	Standard Care	Crude OR (95% CI)	Adjusted OR^a^ (95% CI)
All deliveries = 13,115	*n* = 2679 % (n)	*n* = 10,436 % (n)		
Elective Cesarean Section *n* = 1020	8.4 (225)	7.6 (795)	1.11 (0.95;1.30)	1.02 (0.86;1.21)
Planned vaginal birth *n* = 12,095	*n* = 2454 % (n)	*n* = 9641 % (n)	Crude OR (95% CI)	Adjusted OR^a^ (95% CI)
Birth< 32 weeks	0.7 (17)	1.0 (98)	0.68 (0.41;1.14)	0.71 (0.40;1.24)
Births< 37 weeks	6.9 (168)	6.6 (639)	1.04 (0.87;1.23)	1.10 (0.89;1.36)
Induction	26.0 (639)	25.8 (2484)	1.01 (0.92;1.12)	0.99 (0.88;1.12)
Cervix≤4 cm on arrival	70.1 (520)	73.0 (2572)	0.87 (0.73;1.03)	0.96 (0.80;1.16)
Augmentation (syntocinon)	22.1 (542)	21.8 (2106)	1.01 (0.91;1.13)	1.20 (1.06;1.35)
Amniotomy	21.5 (528)	21.5 (2070)	1.00 (0.90;1.12)	1.05 (0.94;1.17)
Epidural (vaginal birth)	24.4 (599)	26.2 (2523)	0.91 (0.82;1.01)	0.97 (0.86;1.08)
Emergency CS	16.5 (405)	14.5 (1393)	1.17 (1.04;1.32)	1.17 (1.03;1.34)
Instrumental delivery	5.8 (142)	6.5 (631)	0.88 (0.73;1.06)	1.01 (0.83;1.23)
Labour length ≤ 10 h	72.8 (1704)	65.6 (6079)	1.41 (1.27;1.56)	1.26 (1.13;1.42)
No laceration	65.8 (1615)	59.8 (5766)	1.29 (1.18;1.42)	1.17 (1.06;1.29)
Laceration 1 or 2	32.1 (788)	37.7 (3635)	0.78 (0.71;0.86)	0.86 (0.77;0.95)
Laceration 3 or 4	2.3 (57)	2.9 (276)	0.81 (0.60;1.08)	1.00 (0.74;1.36)
Apgar≤7 1 min	6.8 (167)	5.4 (518)	1.29 (1.07;1.54)	1.32 (1.09;1.60)
Apgar≤7 5 min	2.0 (48)	1.3 (124)	1.53 (1.09;2.14)	1.57 (1.11;2.23)
Umb.ven.pH ≤ 7.05	0.5 (11)	0.5 (43)	1.01 (0.52;1.95)	1.02 (0.50;2.07)
Umb.art.pH ≤ 7.05	1.6 (40)	1.5 (145)	1.09 (0.76;1.54)	1.21 (0.84;1.75)
Transfer to NCU	6.2 (151)	5.5 (533)	1.12 (0.93;1.35)	1.20 (0.97;1.47)
Early discharge	33.0 (809)	30.2 (2908)	1.14 (1.04;1.25)	1.03 (0.91;1.16)

^a^Adjusted for maternal age, parity, maternal pre-pregnancy BMI, birth weight, smoking habits, need for interpreter, maternity unit, and birth year. We also controlled for pre-pregnancy risks which included: previous IUGR, caesarean sections, and preterm births. And complications during pregnancy which included: malformations; alcohol or drug abuse; IVF; primiparous< 20; preeclampsia; hypertension; diabetes; premature contractions < 37 weeks of gestation; vaginal bleeding < 37 weeks of gestation; placental abnormalities; uterine abnormalities, and blood type incompatibilities (Rh, ABO, platelets, hydrops foetalis, and other kinds of blood type incompatibilities)

**Table 3:** Duration of labour and hospital stay in caseload midwifery and standard care

	Caseload midwifery *n* = 2679			Standard care *n* = 10,436				
	Mean	(SD)	n	Mean	(SD)	n	Crude mean diff. (95% CI)	Adj.^a^ mean diff. (95% CI)
Duration of labour (minutes)	500	(401)	2338	554	(416)	9251	−55 (−74;-37)	−28 (−45;-10)
Duration of hospital stay after birth (hours)	48.3	(49.1)	2454	53.3	(53.0)	9641	−5.0 (−7.2;-2.8)	−1.5 (−3.2;0.2)

^a^Adjusted for maternal age, parity, maternal pre-pregnancy BMI, birth weight, smoking habits, need for interpreter, maternity unit, and birth year. We also controlled for Pre-pregnancy risks which included: previous IUGR, saesarean sections, and preterm births. And complications during pregnancy which included: malformations; alcohol or drug abuse; IVF; primiparous< 20; preeclampsia; hypertension; diabetes; premature contractions < 37 weeks of gestation; vaginal bleeding < 37 weeks of gestation; placental abnormalities; uterine abnormalities, and blood type incompatibilities (Rh, ABO, platelets, hydrops foetalis, and other kinds of blood type incompatibilities)

Among infants in the caseload group, the adjusted odds for Apgar ≤7 after 1 min were increased (adjusted OR 1.32 (95% CI 1.09–1.60)), as well as the odds for Apgar ≤7 after 5 min (adjusted OR 1.57 (95% CI 1.11–2.23)). The association for low umbilical arterial pH (≤ 7.05) pointed in the same direction, but was much weaker (adjusted OR 1.21 (95% CI 0.84–1.75) *p*-value = 0.31) and the number of infants with low umbilical venous pH (≤ 7.05) did not differentiate between the groups (adjusted OR 1.02 (95% CI 0.50–2.07), *p*-value = 0.96). There was weak evidence for an increase in odds for transfer to the NCU among caseload infants (adjusted OR 1.20 (95% CI 0.97–1.47) *p*-value = 0.08).

### Supplementary analyses

We compared women in caseload midwifery (*n* = 2679) with women in the standard care group with similar transport time to the hospital (*n* = 1236) and found no difference in emergency caesarean section (aOR 1.04; 95% CI 0.84–1.28). We observed more augmentation in the caseload group (aOR 1.28; 95% CI 1.05–1.55) and more infants with low Apgar score at one (aOR 1.43; 1.04–1.98) and five minutes (aOR 2.00; 95% CI 1.02–3.89) in the caseload group (Table 4). All other comparisons including umbilical pH immediately after birth, and transfer to the Neonatal Care Unit (NCU) showed no difference between the two groups (Table 4).

**Table 4:** Labour outcomes in caseload midwifery compared to women with similar distance from their local antenatal clinic to maternity unit

All deliveries = 3915	Caseload Midwifery *n* = 2679% (n)	Similar distance standard care *n* = 1236% (n)	Crude OR (95% CI)	Adj. OR^a^ (95% CI)
Elective Cesarean Section = 341	8.4 (225)	9.4 (116)	0.89 (0.70;1.12)	0.89 (0.69;1.16)
Planned vaginal birth *n* = 3574				
Birth< 32 weeks	0.7 (17)	1.0 (11)	0.70 (0.33;1.51)	0.63 (0.28;1.43)
Births< 37 weeks	6.9 (168)	7.3 (82)	0.93 (0.70;1.23)	0.95 (0.68;1.32)
Induction	26.0 (639)	26.2 (293)	1.07 (0.88;1.30)	1.04 (0.85;1.28)
Cervix ≤4 cm at arrival	70.1 (520)	70.6 (293)	0.98 (0.75;1.27)	0.94 (0.71;1.26)
Augmentation (syntocinon)	22.1 (542)	17.9 (200)	1.30 (1.09;1.56)	1.28 (1.05;1.55)
Amniotomy	21.5 (528)	18.9 (212)	1.17 (0.98;1.40)	1.15 (0.95;1.39)
Epidural (vaginal birth)	24.4 (599)	23.6 (264)	1.05 (0.88;1.24)	1.01 (0.84;1.21)
Emergency CS	16.5 (405)	16.8 (188)	0.98 (0.81;1.19)	1.04 (0.84;1.28)
Instrumental delivery	5.8 (142)	5.9 (66)	0.98 (0.73;1.32)	1.05 (0.77;1.44)
Labour length ≤ 10 h	72.8 (1704)	69.0 (729)	1.20 (1.02;1.41)	1.30 (1.09;1.55)
No laceration	65.8 (1615)	67.0 (750)	0.95 (0.82;1.11)	1.02 (0.87;1.20)
Laceration 1 or 2	32.1 (788)	31.3 (350)	1.04 (0.89;1.21)	0.97 (0.82;1.14)
Laceration 3 or 4	2.3 (57)	1.9 (21)	1.24 (0.75;2.07)	1.21 (0.67;1.87)
Apgar≤7 at 1 min	6.8 (167)	4.9 (55)	1.41 (1.03;1.93)	1.43 (1.04;1.98)
Apgar≤7 at 5 min	2.0 (48)	1.1 (12)	1.84 (0.97;3.48)	2.00 (1.02;3.89)
Umb.ven.pH ≤ 7.05	0.5 (11)	0.5 (6)	0.84 (0.31;2.26)	0.93 (0.34;2.52)
Umb.art.pH ≤ 7.05	1.6 (40)	1.1 (12)	1.53 (0.80;2.93)	1.59 (0.81;3.15)
Transfer to NCU	6.2 (151)	5.9 (66)	1.05 (0.78;1.41)	1.00 (0.73;1.38)
Early discharge	33.0 (809)	31.1 (348)	1.09 (0.94;1.27)	1.05 (0.86;1.28)

^a^Adjusted for maternal age, parity, maternal pre-pregnancy BMI, birth weight, smoking habits, need for interpreter, maternity unit, and birth year. We also controlled for pre-pregnancy risks which included: former IUGR, caesarean sections, and preterm births. And complications during pregnancy included: malformations; alcohol or drug abuse; IVF; primiparous < 20; preeclampsia; hypertension; diabetes; premature contractions < 37 weeks of gestation; vaginal bleeding < 37 weeks of gestation; placental abnormalities; uterine abnormalities, and blood type incompatibilities (Rh, ABO, platelets, hydrops foetalis, and other kinds of blood type incompatibilities)

We repeated the adjusted analyses restricted to the last 14 months of the study period and added adjustment for years in school and level of education (Additional file 1: Table S1). Controlling for these factors had no effect on the odds ratios. Also, differences across the two groups were smaller for this time period.

When restricting the analyses to only low risk women (caseload women compared to standard care women), there was no longer statistically significant evidence for a difference in emergency caesarean or low Apgar score after 5 min, but the results pointed in the same direction as for the main analysis (Additional file 2: Table S2). Low risk was defined as the absence of “pre-pregnancy risks”, “risk factors/complications in the present pregnancy”, BMI < 17, BMI > 30, use of interpreter, and previous spontaneous abortions > 3.

Separate analyses of multiparous and primiparous women showed that the increased OR for augmentation in the caseload group, observed in the main analysis, was only present for multiparous women (adjusted OR 1.49 (95% CI 1.24–1.80)) as compared to primiparous women (adjusted OR 1.05 (95% CI 0.90–1.21)) (Additional file 3: Table S3). Also, the increased OR for both emergency caesarean section and low Apgar score were mainly explained by higher OR among caseload multiparous women as the OR among primiparous women was more modest. In contrast, the increased OR for transfer to the NCU was only present in infants of primiparous women.

When comparing outcomes of caseload women and standard care women separately for each maternity unit, we found that results for augmentation, emergency cesarean section, and low Apgar score were overall comparable to those for the full study group (Additional file 4: Table S4). In maternity unit B, the use of amniotomy was higher in the caseload group and the perineal outcome was slightly better, whereas in maternity unit A, transfer to the NCU was higher in the caseload group.

The sensitivity analyses showed almost no difference according to outcome when homebirths or women who needed an interpreter were excluded from the study population (Additional file 5: Table S5 and Additional file 6: Table S6, respectively). All supplementary tables are available at the homepage.

## Discussion

We hypothesised that this study might find either no difference or only small differences in labour outcomes in favor of caseload midwifery because both models of care in this Danish setting are midwife-led care. We found that women in the caseload group had a shorter duration of labour and received more augmentation, indicating a more active approach to childbirth. In the caseload group, we also found more infants with low Apgar scores. These differences were mainly explained by higher risks in multiparous women. Except for a decreased risk of 1st and 2nd degree lacerations among caseload women, we observed no other differences in labour outcomes between the two groups.

The crude risks of interventions in both our study groups were in general lower than those of other studies (Comparable lists are provided in Additional file 7: Table S7). Midwife-led care in Denmark in general strives at supporting natural and spontaneous childbirth and this approach was emphasized during the study period by several projects at a national level [20–22]. Only one continuous midwife during labour is regarded as preferable. The mean number of midwives per labour was 1.3 in caseload midwifery and 1.8 in standard care. Moreover, respectively 95 and 82% of women in caseload midwifery and standard care saw only two midwives during their labour and birth. These numbers underline that continuity of care during labour is highly prioritized in Denmark, which might impair comparisons of Danish caseload midwifery to international settings.

In both maternity units, caseload midwifery and standard care had equal contexts as they had the same technical equipment, obstetric service and followed the same clinical guidelines. Thus, the main difference between the two models of care was to be ‘known’ to the woman prior to labour. In the literature, it has been difficult to identify studies where the only difference between two midwife-led models of care was continuity of care by the same midwife(ves) across pregnancy, labour and birth. Observational studies reported mainly beneficial or no adverse outcomes when different models of midwife-led continuity of care models were compared with mixed-carer models [12, 23–25] or midwife-led models placed in different settings [26, 27]. In contrast to other studies, our caseload women did not present a selected low-risk population [2, 12, 13, 24, 26, 27], neither were they self-selected into this model of care which is in contrast to other observational studies [1, 23]. This might partly explain why our findings differ from those of previous observational studies [12, 23–27]. Several randomised controlled trials (RCTs) and the Cochrane systematic review “Midwife-led continuity of care models” all confirmed that outcomes of midwife-led models of care were better or had no adverse effects compared to other models of care [1–3, 13]. The contrast to our findings might again be explained by differences in the compared models of care, but weaknesses of the observational design also need to be carefully considered as will be discussed in the following.

Women in the caseload group were of higher parity and slightly heavier, but these factors were adjusted for, as were a number of other potential confounders, chosen a priori. Because allocation to caseload midwifery was geographically determined, we collected information in the last year of the study period about the woman’s years in school and level of education. Including this information in a sub-analysis did not influence the estimates.

Two centers for refugees were in the geographical uptake area of caseload midwifery, and it is known that refugee women have more adverse pregnancy outcomes [28–30]. Excluding women who needed an interpreter (refugees) from the analyses did not change the results. It is known that women with longer transport time to hospital have poorer neonatal outcomes [18], however we did not have information about the addresses of the women. Instead, we defined a group of women in standard care women where the distance from their local antenatal clinics to the hospital was similar to that of the women in caseload midwifery. These women were compared to the caseload women and we found that there was no difference in emergency caesarean sections wheras the differences in augmentation and low Apgar score remained unchanged. We find it likely that the increase in emergency caesarean sections can be explained by the distance to hospital.

In all comparisons, Apgar scores both at 1 and 5 min were less favorable in caseload midwifery. The umbilical arterial pH measures taken immediately after birth and the transfer to NCU pointed in the same direction but showed weaker associations with model of care. The observed absolute differences were small but the severity of these outcomes is important. A recently published study from New Zealand also observed poorer perinatal outcome with caseload midwifery [31], but this study was limited to births after 37 weeks of gestation, and it was not possible to distinguish the model of care received during labour. Also, this study did not take into account the distance to hospital [32].

We observed a higher use of augmentation in the caseload group, and this is in accord with the M@ngo Study which included and randomized all-risk women [1] and in that sense is comparable to the present study. In the M@ngo Study, the neonatal outcome did not differ between the groups [1]. Yet, inappropriate use of syntocinon augmentation is found to lead to adverse perinatal outcome [33–38]. Therefore, low Apgar scores and shorter labours in the caseload group in this study might in part be explained by a higher number of women receiving syntocinon augmentation. Findings from two qualitative studies about the experiences of the midwives [11] and the couples [39], carried out in the same setting and time period, might partly explain these findings. In caseload midwifery, the philosophy of care was found to be one of shared decision making between the women and their caseloading midwives. In addition, the midwives felt very obligated to “be there” for all their women [11]. There is a risk that this approach may lead the midwives to hasten labour, particularly because the couples said that they preferred quick births [39].

Denmark has a long tradition of public registration [40], and register data is known to be a valuable tool for research [41, 42] but is also susceptible to errors [41–44]. The registered codes in the obstetric database were not used in exactly the same way in maternity unit A and B. We contacted both departments to be able to interpret the codes correctly, and our results agreed with the yearly report from each maternity unit. All data were entered into the obstetric database by the midwives during the woman’s pregnancy and childbirth, and any misclassification of these data is most likely to be equally distributed in the two groups.

The study period was limited to three years to ensure that all of the caseload models had been working for at least one year. The statistical power was sufficient to investigate primary outcomes, but in the stratified analyses, and when investigating rare outcomes, estimates had wide confidence intervals due to too few observations. We did not adjust for multiple testing but we were cautionus in our interpretation of the results.

A potential source of confounding could be that some of the unmeasured baseline characteristics differ.

### Generalisability

In most published observational studies, women actively chose to join a midwife-led model of care [12, 24, 26, 27, 31] and these models only included women at low risk [12, 13, 24, 26, 27]. In the present study, caseload midwifery was geographically allocated and women at all risks were included. The intervention rate in both midwife-led models was low and continuity of care was also found in standard care. The generalisability of our findings outside Denmark might therefore be difficult and needs further consideration.

## Conclusion

We hypothesised that this study might find either no difference or only small differences in birth outcomes in favor of caseload midwifery. We found that most outcomes were equal across models of care but there seemed to be a small but unexpected finding of more augmentation and adverse neonatal outcomes in caseload midwifery. These findings should be interpreted in the context of the overall low intervention and complication rate in this Danish setting and in the context of research that supports the benefits of caseload midwifery. Also, the observational design of the study allows only cautious conclusions.

### Implications for practice

These findings underline it is important to monitor and evaluate new models of care in a local setting even though international, robust evidence points to a positive effect. Two midwife-led models were investigated and the only difference between these models seemed to be the model of care as all other practical and organisational circumstances were equal. There is a need to understand how or if caseload midwifery in Denmark influences labour outcomes differently than seen in most international studies. Therefore an evaluation of other models of caseload midwifery in Denmark is needed.

### Impact on local practice

The results suggests the possibility of inadvertent harm when midwives and women are very keen on ensuring that the primary midwife is present at the birth. Therefore, the findings of this study have led to the following changes in the caseloading model in Maternity Unit A, and changes in Maternity Unit B will follow. The number of midwives in a caseload has been changed; previously, midwives mainly worked in pairs, now there are most often three midwives in each caseload. Days on call have been changed from 7 days to 3–4 days. Finally, working hours have changed. Previously, there was no excact limit. Now after 12 h, the midwife should discuss with her colleagues whether she should stay. After 16 h, she is expected to go home to rest.

## Additional files

Additional file 1: **Table S1.** Labour outcomes in caseload midwifery and standard care - adjusted for years in school and the level of professional education (time: 1/11 2014–31/12 2015). (DOCX 23 kb)

Additional file 2: **Table S2.** Labour outcomes in caseload midwifery and standard care - only low risk women. (DOCX 22 kb)

Additional file 3: **Table S3.** Labour outcomes in caseload midwifery and standard care - stratified by primi- and multiparous. (DOCX 25 kb)

Additional file 4: **Table S4.** Labour outcomes in caseload midwifery and standard care - stratified by maternity units. (DOCX 24 kb)

Additional file 5: **Table S5.** Labour outcomes in caseload midwifery and standard care - homebirth excluded. (DOCX 21 kb)

Additional file 6: **Table S6.** Labour outcomes in caseload midwifery and standard care - women in need of an interpreter are excluded. (DOCX 22 kb)

Additional file 7: **Table S7.** Labour outcomes in caseload midwifery and standard care - Outcomes in the present study and findings from nine different international studies. (DOCX 37 kb)

## Abbreviations

aOR: Adjusted Odds Ratio
ICD: International Classification of Diseases
NCU: Neonatal Care Unit
BMI: Body Mass Index
SD: Standard Deviation
RCT: Randomised Controlled Trial

## References

[1] Tracy SK, Hartz DL, Tracy MB, Allen J, Forti A, Hall B (2013). Caseload midwifery care versus standard maternity care for women of any risk: M@NGO, a randomised controlled trial. Lancet.

[2] McLachlan HL, Forster DA, Davey MA, Farrell T, Gold L, Biro MA (2012). Effects of continuity of care by a primary midwife (caseload midwifery) on caesarean section rates in women of low obstetric risk: the COSMOS randomised controlled trial. BJOG.

[3] Sandall J, Soltani H, Gates S, Shennan A, Devane D. Midwife-led continuity models versus other models of care for childbearing women. Cochrane Database Syst Rev. 2016. 10.1002/14651858.CD004667.pub5. 10.1002/14651858.CD004667.pub5 PMC8663203 27121907

[4] Homer C, Leap N, Brodie P. Midwifery continuity of care: a practical guide. Sydney, N.S.W: Churchill Livingstone; 2008.

[5] Soltani H, Sandall J (2012). Organisation of maternity care and choices of mode of birth: a worldwide view. Midwifery.

[6] Møller J, Cliff H, Larsen M, Blinkenberg K. Beskrivelse af jordemoderområdet: Bind 1 [Description of the area of midwifery: vol. 1]. København [Copenhage]: Den Almindelige Danske Jordemoderforening [Danish Midwifery Union]; 1997.

[7] ICM International Definition of the Midwife. International Confederation of Midwives. 2011. https://www.internationalmidwives.org/our-work/policy-and-practice/icm-definitions.html. Accessed 22 Nov 2018.

[8] National Board of Health. Circular of Midwifery. Circular No. 149, 2001. http://www.sst.dk/publ/Vejledninger/01/149.pdf. Accessed 22 Nov 2018.

[9] Jordemoderliv CH (1992). The life of a midwife.

[10] Til gravide i Himmerland [To pregnant women living in Himmerland] https://aalborguh.rn.dk/afsnit-og-ambulatorier/gynaekologisk-obstetrisk-afdeling-aalborg/undersoegelser-og-behandlinger/gynaekologi-graviditet-og-foedsel?rnid=kbua02-119. Accessed 22 Nov 2018.

[11] Jepsen I, Mark E, Nohr EA, Foureur M, Sorensen EE (2016). A qualitative study of how caseload midwifery is constituted and experienced by Danish midwives. Midwifery.

[12] Beckmann M, Kildea S, Gibbons K (2012). Midwifery group practice and mode of birth. Women and Birth.

[13] Begley C, Devane D, Clarke M, McCann C, Hughes P, Reilly M, et al. Comparison of midwife-led and consultant-led care of healthy women at low risk of childbirth complications in the Republic of Ireland: a randomised trial. BMC Pregnancy Childbirth. 2011. 10.1186/1471-2393-11-85. 10.1186/1471-2393-11-85 PMC3226589 22035427

[14] Biró M, Waldenström U, Pannifex JH (2000). Team midwifery care in a tertiary level obstetric service: a randomized controlled trial. Birth.

[15] Løvschall C, Burau V, Søgaard R, Holst A, Lyngsø C, Fogsgaard A (2013). Evaluering af kendt jordemoderordning [evaluation of caseload midwifery].

[16] Forster DA, Newton M, McLachlan HL, Willis K (2011). Exploring implementation and sustainability of models of care: can theory help?. BMC Public Health.

[17] Classifications. 2016. http://sundhedsdatastyrelsen.dk/da/rammer-og-retningslinjer/om-klassifikationer. Accessed 22 Nov 2018.

[18] Ravelli A, Jager KJ, de Groot M, Erwich J, Rijninks-van Driel G, Tromp M (2011). Travel time from home to hospital and adverse perinatal outcomes in women at term in the Netherlands. BJOG Int J Obstet Gynaecol.

[19] StataCorp (2013). Stata Statistical Software: Release 13. College Station.

[20] Kjeldset A, Rischel V, Larson MO, Munk L, Villadsen MY (2013). Sikre fødsler. Et skridt ad gangen. [Safe births. One step at a time]. Tidsskrift for jordemødre.

[21] Kjeldsen A (2014). Vi skal turde være tålmodige [We have to dare to be patient]. Tidsskrift for jordemødre..

[22] Forebyggelse af Sphinkter ruptur [Prevention of anal sphinkter rupture]. 2015.http://clin.au.dk/fileadmin/www.ki.au.dk/forskning/forskningsenheder/gyn__kologisk-obstetrisk_afd__y/logistics/sandbjerg_m__der/Sandbjerg_2015/Forebyggelse_af_sfincterruptur_med_evidenstabeller.pdf. Accessed 22 Nov 2018.

[23] Page L, McCourt C, Beake S, Vail A, Hewison J (1999). Clinical interventions and outcomes of one-to-one midwifery practice. J Public Health.

[24] Tracy SK, Welsh A, Hall B, Hartz D, Lainchbury A, Bisits A, et al. Caseload midwifery compared to standard or private obstetric care for first time mothers in a public teaching hospital in Australia: a cross sectional study of cost and birth outcomes. BMC Pregnancy and Childbirth. 2014. 10.1186/1471-2393-14-46. 10.1186/1471-2393-14-46 PMC3903023 24456576

[25] Johnson M, Stewart H, Langdon R, Kelly P (2005). Yong L. a comparison of the outcomes of partnership caseload midwifery and standard hospital care in low risk mothers. Aust J Adv Nurs.

[26] Eide BI, Nilsen ABV, Rasmussen S. Births in two different delivery units in the same clinic - a prospective study of healthy primiparous women. BMC Pregnancy and Childbirth. . 10.1186/1471-2393-9-25. 10.1186/1471-2393-9-25 PMC2712449 19545412

[27] Gottvall K, Waldenström U, Tingstig C, Grunewald C (2011). In-hospital birth center with the same medical guidelines as standard care: a comparative study of obstetric interventions and outcomes. Birth.

[28] Gibson-Helm M, Boyle J, Cheng I, East C, Knight M, Teede H (2015). Maternal health and pregnancy outcomes among women of refugee background from Asian countries. Int J Gynecol Obstet.

[29] Malin M, Gissler M. Maternal care and birth outcomes among ethnic minority women in Finland. BMC Public Health. 2010. 10.1136/bmj.c1471. 10.1186/1471-2458-9-84 PMC2674879 19298682

[30] Troe EWM, Bos V, Deerenberg IM, Mackenbach JP, Joung IMA (2006). Ethnic differences in total and cause-specific infant mortality in the Netherlands. Paediatr Perinat Epidemiol.

[31] Wernham E, Gurney J, Stanley J, Ellison-Loschmann L, Sarfati D. A comparison of midwife-led and medical-led models of care and their relationship to adverse fetal and neonatal outcomes: a retrospective cohort study in New Zealand. PLoS Med. 2016. 10.1371/journal.pmed.1002134. 10.1371/journal.pmed.1002134 PMC5038958 27676611

[32] de Jonge A, Sandall J (2016). Improving research into models of maternity care to inform decision making. PLoS Med.

[33] Hayes EJ, Weinstein L. Improving patient safety and uniformity of care by a standardized regimen for the use of oxytocin. Obstet Gynecol 2008;198:622.e1-e7. 10.1016/j.ajog.2008.01.039 18355786

[34] Berglund S, Grunewald C, Pettersson H, Cnattingius S (2008). Severe asphyxia due to delivery-related malpractice in Sweden 1990–2005. BJOG Int J Obstet Gynaecol.

[35] Selin L, Almström E, Wallin G, Berg M (2009). Use and abuse of oxytocin for augmentation of labor. Acta Obstet Gynecol Scand.

[36] Hove LD, Bock J, Christoffersen JK, Hedegaard M (2008). Analysis of 127 peripartum hypoxic brain injuries from closed claims registered by the Danish patient insurance association. Acta Obstet Gynecol Scand.

[37] Jonsson M, Lindeberg SN, Hanson U (2007). Analysis of malpractice claims with a focus on oxytocin use in labour. Acta Obstet Gynecol Scand.

[38] Clark K. Midwifery first year of practice, information booklet 2016. New Zealand College of Midwives. 2016.

[39] Jepsen I, Mark E, Foureur M, Nohr EA. Sorensen EE. A qualitative study of how caseload midwifery is experienced by couples in Denmark. Women Birth. 2017. 10.1016/j.wombi.2016.09.003 27665216

[40] Frank L. When an Entire Country Is a Cohort. Science. 2000;287:2398–9.42 10.1126/science.287.5462.2398 10766613

[41] Lynge E, Sandegaard JL, Rebolj M. The Danish National Patient Register. Scandinavian Journal of Public Health 2011;39:30–3. 10.1177/1403494811401482 21775347

[42] Schmidt M, Schmidt SAJ, Sandegaard JL, Ehrenstein V, Pedersen L, Sørensen HT (2015). The Danish national patient registry: a review of content, data quality, and research potential. Clin Epidemiol.

[43] Lass P, Lilholt J, Thomsen L, Lundbye-Christensen S, Enevoldsen H, Simonsen OH (2006). The quality of diagnosis and procedure coding in orthopaedic surgery northern Jutland. Ugeskr Laeg.

[44] Gissler M, Shelley J (2002). Quality of data on subsequent events in a routine medical birth register. Medical Information Internet in Med.

[45] Guidelines about Notification etc. of a Biomed Res Project to the Committee System on Biomed Res Ethics, No 9154, 5 May 2011. http://www.nvk.dk/~/media/NVK/Dokumenter/Vejledning_Engelsk.pdf. Accessed 22 Nov 2018.

