# Supplementary material for: Labour outcomes in caseload midwifery and standard care: a register-based cohort study
Source: BMC Pregnancy Childbirth. 2018 Dec 6;18:481. doi: 10.1186/s12884-018-2090-9 (PMC6282374; doi:10.1186/s12884-018-2090-9)
Supplement: Supplementary file 1 — Table S1. Labour outcomes in caseload midwifery and standard care - adjusted for years in school and the level of professional education (time: 1/11 2014–31/12 2015). (DOCX 23 kb) [file 12884_2018_2090_MOESM1_ESM.docx]

Table S1). Labour outcomes in caseload midwifery and standard care - adjusted for years in school and the level of professional education (time: 1/11 2014 - 31/12 2015)

|  | Analysis I*  Adjusted for a priori chosen confounders | Analysis II**  Further adjusted for school and education |
| --- | --- | --- |
|  | Adj. OR* (95% CI) | Adj. OR** (95% CI) |
| Elective CS (n=388) | 1.18 (0.90;1.56) | 1.19 (0.90:1.57) |
| Planned vaginal birth (n=4427) |  |  |
| Birth<32 weeks | 0.78 (0.30;2.05) | 0.78 (0.29;2.05) |
| Births<37 weeks | 0.90 (0.61;1.32) | 0.90 (0.61;1,32) |
| Induction | 0.98 (0.79;1.20) | 0.97 (0.79;1.19) |
| Cervix ≤4cm at arrival | 0.96 (0.80:1.16) | 0.96 (0.80;1.16) |
| Augmentation (syntocinon) | 1.11 (0.91;1.36) | 1.11 (0.90;1.36) |
| Amniotomy | 1.22 (1.01;1.47) | 1.22 (1.01;1.47) |
| Epidural (vaginal birth) | 0.97 (0.80;1.18) | 0.97 (0.80;1.18) |
| Emergency CS | 1.16 (0.91;1.47) | 1.15 (0.91;1.46) |
| Instrumental delivery | 1.26 (0.91;1.74) | 1.26 (0.91;1.75) |
| Labour length≤10 hours | 1.34 (1.10; 1.63) | 1.34 (1.10; 1.63) |
| No laceration | 1.17 (0.99;1.38) | 1.16 (0.98;1.37) |
| Laceration 1 or 2 | 0.85 (0.72;1.00) | 0.85 (0.72;1.01) |
| Laceration 3 or 4 | 0.94 (0.57;1.55) | 0.94 (0.57;1.55) |
| Apgar≤7 1. minute | 1.38 (1.01;1.88) | 1.36 (0.99;1.85) |
| Apgar≤7 5. minute | 1.36 (0.72;2.57) | 1.33 (0.70;2.51) |
| Umb.ven.pH≤7.05 | 0.71 (0.16;3.23) | 0.71 (0.16;3.23) |
| Umb.art.pH≤7.05 | 1.24 (0.67;2.29) | 1.23 (0.66;2.27) |
| Transfer to NCU | 1.11 (0.77;1.61) | 1.08 (0.75;1.57) |
| Early discharge | 1.12 (0.91:1.39) | 1.13 (0.91:1.39) |

*Confounders analysis I: maternal age, parity, maternal pre-pregnancy BMI, birth weight, smoking habits, need for interpreter, maternity unit, and birth year. We also controlled for pre-pregnancy risks which included: previous IUGR, caesarean sections, and preterm births. And complications during pregnancy which included: malformations; alcohol or drug abuse; IVF; primiparous<20; preeclampsia; hypertension; diabetes; premature contractions < 37 weeks of gestation; vaginal bleeding <37 weeks of gestation; placental abnormalities; uterine abnormalities, and blood type incompatibilities (Rh, ABO, platelets, hydrops foetalis, and other kinds of blood type incompatibilities).

** Confounders analysis II: Years in school and level of education was added to the list of confounders in analysis I.
