# Supplementary material for: Labour outcomes in caseload midwifery and standard care: a register-based cohort study
Source: BMC Pregnancy Childbirth. 2018 Dec 6;18:481. doi: 10.1186/s12884-018-2090-9 (PMC6282374; doi:10.1186/s12884-018-2090-9)
Supplement: Supplementary file 2 — Table S2. Labour outcomes in caseload midwifery and standard care - only low risk women. (DOCX 22 kb) [file 12884_2018_2090_MOESM2_ESM.docx]

Table S2) Labour outcomes in caseload midwifery and standard care - only low risk women

|  | Caseload  Midwifery  n= 2679  % (n) | Standard  Care  n=10436  % (n) | Crude  OR (95% CI) | Adj.  OR* (95% CI) |
| --- | --- | --- | --- | --- |
| All deliveries=13115 |  |  |  |  |
| Elective Cesarean Section  n=1020 | 3.0 (38) | 3.2 (169) | 0.94 (0.66;1.34) | 0.86 (0.59;1.25) |
| Planned vaginal birth n=12095 |  |  |  |  |
| Birth<32 weeks | 0.3 (3) | 0.4 (19) | 0,66 (0.19;2.23) | 0.38 (0.08;1.87) |
| Births<37 weeks | 5.9 (72) | 4.4 (225) | 1.36 (1.03;1.78) | 1.36 (0.99;1.86) |
| Induction | 12.4 (151) | 11.7 (594) | 1.07 (0.88;1.30) | 1.04 (0.85;1.28) |
| Cervix ≤4cm at arrival | 61.6 (245) | 65.7 (1253) | 0.83 (0.67;1.04) | 0.94 (0.74;1.19) |
| Augmentation (syntocinon) | 20.9 (255) | 20.5 (1043) | 1.03 (0.88;1.20) | 1,25 (1.05;1.48) |
| Amniotomy | 23.7 (288) | 22.7 (1155) | 1.05 (0.91;1.22) | 1.10 (0.95;1.28) |
| Epidural (vaginal birth) | 20.0 (244) | 21.4 (1086) | 0.92 (0.79;1.08) | 1.01 (0.86;1.20) |
| Emergency CS | 9.4 (115) | 8.4 (427) | 1.14 (0.92;1.41) | 1.09 (0.86;1.38) |
| Instrumental delivery | 6.7 (82) | 6.8 (346) | 0.99 (0.77;1.27) | 1.12 (0.85;1.46) |
| Labour length≤10 hours | 71.7 (863) | 64.3 (3219) | 1.40 (1.22;1.61) | 1.22 (1.04;1.43) |
| No laceration | 64.6 (787) | 57.3 (2911) | 1.36 (1.19;1.55) | 1.22 (1.06;1.40) |
| Laceration 1 or 2 | 33.1 (403) | 40.1 (2038) | 0.74 (0.65;0.84) | 0.82 (0.71;0.94) |
| Laceration 3 or 4 | 2.5 (30) | 2.9 (149) | 0.84 (0.56;1.24) | 0.98 (0.65;1.49) |
| Apgar≤7 1. minute | 5.0 (61) | 4.0 (201) | 1.28 (0.95;1.72) | 1.42 (1.04;1.94) |
| Apgar≤7 5. minute | 1.2 (14) | 1.0 (49) | 1.19 (0.66;2.17) | 1.43 (0.75;2.71) |
| Umb.ven.pH≤7.05 | 0.4 (5) | 0.4 (21) | 0.99 (0.37;2.64) | 1.23 (0.45;3.40) |
| Umb.art.pH≤7.05 | 1.7 (21) | 1.4 (71) | 1.24 (0.76;2.02) | 1.50 (0.89;2.54) |
| Transfer to NCU | 4.6 (56) | 3.4 (175) | 1.35 (0.99;1.84) | 1.45 (1.04;2.04) |
| Early discharge | 37.7 (459) | 34.3 (1744) | 1.16 (1.02;1.32) | 1.09 (0.90;1.31) |

*Adjusted for maternal age, parity, maternal pre-pregnancy BMI, birth weight, smoking habits, need for interpreter, maternity unit, and birth year. We also controlled for pre-pregnancy risks which included: previous IUGR, caesarean sections, and preterm births., and for complications during pregnancy which included: malformations; alcohol or drug abuse; IVF; primiparous<20; preeclampsia; hypertension; diabetes; premature contractions < 37 weeks of gestation; vaginal bleeding <37 weeks of gestation; placental abnormalities; uterine abnormalities, and blood type incompatibilities (Rh, ABO, platelets, hydrops foetalis, and other kinds of blood type incompatibilities).
