# Supplementary material for: Labour outcomes in caseload midwifery and standard care: a register-based cohort study
Source: BMC Pregnancy Childbirth. 2018 Dec 6;18:481. doi: 10.1186/s12884-018-2090-9 (PMC6282374; doi:10.1186/s12884-018-2090-9)
Supplement: Supplementary file 3 — Table S3. Labour outcomes in caseload midwifery and standard care - stratified by primi- and multiparous. (DOCX 25 kb) [file 12884_2018_2090_MOESM3_ESM.docx]

Table S3) Labour outcomes in caseload midwifery and standard care - stratified by primi- and multiparous

|  | Primiparous n= 6131 | | Multiparous n=6984 | |
| --- | --- | --- | --- | --- |
|  | Crude OR | Adj. OR* (95% CI | Crude OR | Adj. OR* (95% CI) |
| Number of Elective Cesarean Section | n=249 | | n=771 | |
| Elective Cesarean Section | 0.83 | 0.83 (0.59;1.19) | 1.10 | 1.08 (0.88;1.32) |
| Number of Planned vaginal birth | n=5882 | | n=6213 | |
| Birth<32 weeks | 0.73 | 0.82(0.38;1.76) | 0.62 | 0.59(0.26;1.34) |
| Births<37 weeks | 0.96 | 0.98 (0.73;1.32) | 1.08 | 1.17 (0.86;1.57) |
| Induction | 0.96 | 0.94 (0.79;1.12) | 1.07 | 1.05 (0.89;1.22) |
| Cervix ≤4cm at arrival | 0.73 | 0.81 (0.61;1.08) | 1.03 | 1.12 (0.88;1.41) |
| Augmentation (syntocinon) | 0.98 | 1.05 (0.90;1.21) | 1.43 | 1.49 (1.24;1.80) |
| Amniotomy | 0.99 | 1.05 (0.89;1.25) | 1.00 | 1.04 (0.90;1.20) |
| Epidural (vaginal birth) | 0.95 | 0.95 (0.82;1.10) | 1.01 | 1.00 (0.85;1.19) |
| Emergency CS | 1.17 | 1.11 (0.93;1.32) | 1.31 | 1.28 (1.04;1.56) |
| Instrumental delivery | 0.99 | 1.02 (0.82;1.27) | 0.90 | 0.98 (0.63;1.52) |
| Birth length≤10 hours | 1.36 | 1.29 (1.12;1.49) | 1.23 | 1.22 (1.02;1.46) |
| No laceration | 1.15 | 1.09 (0.94;1.25) | 1.37 | 1.24 (1.09;1.42) |
| Laceration 1 or 2 | 0.89 | 0.93 (0.81;1.08) | 0.72 | 0.79 (0.69;0.91) |
| Laceration 3 or 4 | 0.78 | 0.90 (0.62;1.29) | 1.15 | 1.27 (0.75;2.17 |
| Apgar≤7 1. minute | 1.18 | 1.18 (0.91;1.54) | 1.42 | 1.35 (1.03;1.78) |
| Apgar≤7 5. minute | 1.34 | 1.43 (0.89;2.29) | 1.84 | 1.69 (1.01;2.83) |
| Umb.ven.pH≤7.05 | 0.49 | 0.55 (0.15;1.97) | 1.62 | 1.42 (0.57;3.56) |
| Umb.art.pH≤7.05 | 0.92 | 1.02 (0.62;1.69) | 1.46 | 1.41 (0.81;2.46) |
| Transfer to NCU | 1.24 | 1.33 (1.01;1.74) | 0.99 | 0.98 (0.74;1.31) |
| Early discharge | 0.94 | 0.94 (0.65;1.35) | 0.95 | 1.01 (0.89;1.15) |

*Adjusted for maternal age, parity, maternal pre-pregnancy BMI, birth weight, smoking habits, need for interpreter, maternity unit, and birth year. We also controlled for pre-pregnancy risks which included: previous IUGR, caesarean sections, and preterm births., and for complications during pregnancy which included: malformations; alcohol or drug abuse; IVF; primiparous<20; preeclampsia; hypertension; diabetes; premature contractions < 37 weeks of gestation; vaginal bleeding <37 weeks of gestation; placental abnormalities; uterine abnormalities, and blood type incompatibilities (Rh, ABO, platelets, hydrops foetalis, and other kinds of blood type incompatibilities).
