# Supplementary material for: Labour outcomes in caseload midwifery and standard care: a register-based cohort study
Source: BMC Pregnancy Childbirth. 2018 Dec 6;18:481. doi: 10.1186/s12884-018-2090-9 (PMC6282374; doi:10.1186/s12884-018-2090-9)
Supplement: Supplementary file 4 — Table S4. Labour outcomes in caseload midwifery and standard care - stratified by maternity units. (DOCX 24 kb) [file 12884_2018_2090_MOESM4_ESM.docx]

Table S4) Labour outcomes in caseload midwifery and standard care - stratified by maternity units

|  | Maternity unit A | | Maternity unit B | |
| --- | --- | --- | --- | --- |
|  | Crude OR | Adj. OR* (95% CI) | Crude OR | Adj. OR* (95% CI) |
| Elective Cesarean Section | 1.23 | 1.13 (0.91;1.40) | 0.88 | 0.85 (0.65; 1.12) |
| Planned vaginal birth  n=(12095) |  |  |  |  |
| Births<37 weeks | 1.12 | 1.07 0.82;1.39) | 1.10. | 1.13 (0.79;1.60) |
| Induction | 1.10 | 1.01 (0.87;1.18) | 1.04 | 0.94 (0.77;1.15) |
| Cervix ≤4cm at arrival | 0.95 | 0.95 (0.74;1.22) | 0.96 | 0.96 (0.73;1.27) |
| Augmentation (syntocinon) | 1.06 | 1.17 (1.01;1.36) | 1.13 | 1.22 (1.00; 1.48) |
| Amniotomy | 0.99 | 0.96 (0.84 ;1.11) | 1.17 | 1.21 (1.00;1.46) |
| Epidural (vaginal birth) | 0.93 | 0.91 (0.79;1.06) | 1.05 | 1.06 (0.88;1.29) |
| Emergency CS | 1.16 | 1.18 (0.99;1.40) | 1.14 | 1.18 (0.95;1.46) |
| Instrumental delivery | 0.84 | 0.97 (0.75;1.26) | 0.98 | 1.06 (0.78;1.45) |
| Labour length≤10 hours | 1.28 | 1.22 (1.05;1.40) | 1.37 | 1.32 (1.10;1.60) |
| No laceration | 1.17 | 1.10 (0.97;1.24) | 1.27 | 1.29 (1.09;1.52) |
| Laceration 1 or 2 | 0.86 | 0.91 (0.80;1.03) | 0.79 | 0.78 (0.66;0.92) |
| Laceration 3 or 4 | 0.91 | 1.10 (0.77;1.57) | 0.83 | 0.90 (0.52;1.56) |
| Apgar≤7 1. minute | 1.43 | 1.43 (1.13;1.81) | 1.12 | 1.14 (0.83;1.56) |
| Apgar≤7 5. minute | 1.55 | 1.45 (0.93;2.27) | 1.58 | 1.70 (0.93;3.09) |
| Umb.ven,pH≤7.05 | 1.34 | 1.47 (0.64;3.39) | 0.57 | 0.58 (0.19;1.76) |
| Umb.artpH≤7.05 | 1.16 | 1.22 (0.78;1.91) | 1.07 | 1.06 (0.55;2.04) |
| Transfer to NCU | 1.42 | 1.39 (1.10;1.77) | 0.80 | 0.80 (0.54;1.19) |
| Early discharge | 1.09 | 0.97 (0.82;1.14) | 1.18 | 1.10 (0.91;1.34) |

*Adjusted for maternal age, parity, maternal pre-pregnancy BMI, birth weight, smoking habits, need for interpreter, maternity unit, and birth year. We also controlled for pre-pregnancy risks which included: previous IUGR, caesarean sections, and preterm births., and for complications during pregnancy which included: malformations; alcohol or drug abuse; IVF; primiparous<20; preeclampsia; hypertension; diabetes; premature contractions < 37 weeks of gestation; vaginal bleeding <37 weeks of gestation; placental abnormalities; uterine abnormalities, and blood type incompatibilities (Rh, ABO, platelets, hydrops foetalis, and other kinds of blood type incompatibilities).
