# Supplementary material for: Labour outcomes in caseload midwifery and standard care: a register-based cohort study
Source: BMC Pregnancy Childbirth. 2018 Dec 6;18:481. doi: 10.1186/s12884-018-2090-9 (PMC6282374; doi:10.1186/s12884-018-2090-9)
Supplement: Supplementary file 5 — Table S5. Labour outcomes in caseload midwifery and standard care - homebirth excluded. (DOCX 21 kb) [file 12884_2018_2090_MOESM5_ESM.docx]

Table S5) Labour outcomes in caseload midwifery and standard care - homebirth excluded

|  | Caseload  Midwifery  % (n) | Standard  Care  % (n) | Crude  OR (95% CI) | Adj.  OR* (95% CI) |
| --- | --- | --- | --- | --- |
| All deliveries=12856 |  |  |  |  |
| Elective Cesarean Section n = 1020 | 8.7 (225) | 7.8 (795) | 1.13 (0.96;1.32) | 1.03 (0.87;1.22) |
| Planned vaginal birth n=11836 | 91.3(2361) | 92.2(9397) |  |  |
| Birth<32 weeks | 0.7 (16) | 1.0 (98) | 0.65 (0.38;1.10) | 0.67 (0.37;1.19) |
| Births<37 weeks | 7.0 (166) | 6.7 (638) | 1.04 (0.87;1.24) | 1.09 (0.88;1.35) |
| Induction | 26.9 (639) | 26.3 (2484) | 1.04 (0.93;1.15) | 1.01 (0.89;1.14) |
| Cervix ≤4cm at arrival | 70.4 (512) | 74.0 (2550) | 0.84 (0.70; 1.00) | 0.93 (0.77;1.12) |
| Augmentation (syntocinon) | 22.8 (542) | 22.3 (2106) | 1.03 (0.93;1.15) | 1.21 (1.07;1.36) |
| Amniotomy | 22.0 (522) | 21.7 (2041) | 1.02 (0.91;1.13) | 1.06 (0.95;1.19) |
| Epidural (vaginal birth) | 25.2 (598) | 26.7 (2523) | 0.93 (0.83;1.03) | 0.98 (0.87;1.09) |
| Emergency CS | 17.1 (405) | 14.7 (1393) | 1.19 (1.05;1.35) | 1.18 (1.03;1.35) |
| Instrumental delivery | 6.0 (142) | 6.7 (631) | 0.89 (0.74;1.07) | 1.01 (0.83;1.23) |
| Labour length≤10 hours | 72.2 (1632) | 65.1 (5921) | 1.39 (1.26;1.54) | 1.27 (1.13; 1.43) |
| No laceration | 65.3 (1549) | 59.8 (5654) | 1.27 (1.15;1.39) | 1.14 (1.03;1.26) |
| Laceration 1 or 2 | 32.6 (773) | 37.7 (3571) | 0.80 (0.72;0.88) | 0.88 (0.79;0.97) |
| Laceration 3 or 4 | 2.4 (57) | 2.9 (274) | 0.83 (0.62;1.11) | 1.02 (0.75;1.38) |
| Apgar≤7 1. minute | 7.0 (165) | 5.4 (514) | 1.30 (1.08;1.56) | 1.32 (1.09;1.60) |
| Apgar≤7 5. minute | 2.0 (48) | 1.3 (124) | 1.55 (1.11;2.18) | 1.58 (1.11;2.24) |
| Umb.ven.pH≤7.05 | 0.5 (11) | 0.5 (43) | 1.02 (0.53;1.98) | 1.03 (0.50;2.10) |
| Umb.art.pH≤7.05 | 1.7 (40) | 1.5 (145) | 1.10 (0.77;1.57) | 1.22 (0.84;1.76) |
| Transfer to NCU | 6.4 (151) | 5.6 (531) | 1.14 (0.95;1.38) | 1.21 (0.99;1.49) |
| Early discharge | 31.1 (739) | 29.2 (2767) | 1.09 (0.99;1.21) | 0.99 (0.87;1.13) |

*Adjusted for maternal age, parity, maternal pre-pregnancy BMI, birth weight, smoking habits, need for interpreter, maternity unit, and birth year. We also controlled for pre-pregnancy risks which included: previous IUGR, caesarean sections, and preterm births., and for complications during pregnancy which included: malformations; alcohol or drug abuse; IVF; primiparous<20; preeclampsia; hypertension; diabetes; premature contractions < 37 weeks of gestation; vaginal bleeding <37 weeks of gestation; placental abnormalities; uterine abnormalities, and blood type incompatibilities (Rh, ABO, platelets, hydrops foetalis, and other kinds of blood type incompatibilities).
