# Supplementary material for: Labour outcomes in caseload midwifery and standard care: a register-based cohort study
Source: BMC Pregnancy Childbirth. 2018 Dec 6;18:481. doi: 10.1186/s12884-018-2090-9 (PMC6282374; doi:10.1186/s12884-018-2090-9)
Supplement: Supplementary file 6 — Table S6. Labour outcomes in caseload midwifery and standard care - women in need of an interpreter are excluded. (DOCX 22 kb) [file 12884_2018_2090_MOESM6_ESM.docx]

Table S6) Labour outcomes in caseload midwifery and standard care - women in need of an interpreter are excluded.

|  | Caseload  Midwifery  % (n) | Standard  Care  % (n) | Crude  OR (95% CI) | Adj.  OR* (95% CI) |
| --- | --- | --- | --- | --- |
| All deliveries=12734 |  |  |  |  |
| Elective Cesarean Section  n= 987 | 8.3 (210) | 7.6 (777) | 1.09 (0.93;1.28) | 1.00 (0.84;1.19) |
| Planned vaginal birth n=11747 |  |  |  |  |
| Birth<32 weeks | 0.7 (16) | 1.0 (95) | 0.68 (0.40;1.15) | 0.69 (0.39;1.24) |
| Births<37 weeks | 7.0 (163) | 6.6 (617) | 1.07 (0.90.;1.28) | 1.13 (0.91;1.41) |
| Induction | 26.0 (606) | 25.9 (2433) | 1.01 (0.91;1.12) | 0.99 (0.87;1.12) |
| Cervix ≤4cm at arrival | 69.7 (497) | 73.2 (2510) | 0.84 (0.71;1.01) | 0.94 (0.77;1.13) |
| Augmentation (syntocinon) | 21.9 (510) | 21.9 (2058) | 1.00 (0.89;1.12) | 1.18 (1.05;1.34) |
| Amniotomy | 21.0 (491) | 21.4 (2016) | 0.98 (0.87;1.09) | 1.03 (0.92;1.16) |
| Epidural (vaginal birth) | 24.6 (573) | 26.3 (2476) | 0.91 (0.82;1.01) | 0.97 (0.86;1.09) |
| Emergency CS | 16.4 (383) | 14.4 (1352) | 1.17 (1.03;1.33) | 1.17 (1.02;1.35) |
| Instrumental delivery | 5.5 (136) | 6.6 (621) | 0.88 (0.72;1.06) | 1.00 (0.82;1.22) |
| Labour length≤10 hours | 73.1 (1628) | 65.8 (5964) | 1.41 (1.27;1.57) | 1.24 (1.11; 1.39) |
| No laceration | 65.9 (1539) | 59.7 (5615) | 1.31 (1.19;1.44) | 1.18 (1.07;1.31) |
| Laceration 1 or 2 | 32.0 (747) | 37.8 (3561) | 0.77 (0.70; 0.85) | 0.85 (0.76;0.94) |
| Laceration 3 or 4 | 2.3 (54) | 2.9 (273) | 0.79 (0.59;1.07) | 0.98 (0.72;1.34) |
| Apgar≤7 1. minute | 6.9 (160) | 5.3 (503) | 1.30 (1.08;1.57) | 1.34 (1.11;1.63) |
| Apgar≤7 5. minute | 2.0 (46) | 1.3 (119) | 1.57 (1.11;2.21) | 1.59 (1.11;2.27) |
| Umb.ven.pH≤7.05 | 0.5 (11) | 0.5 (43) | 1.03 (0.53;2.00) | 1.02 (0.50;2.07) |
| Umb.art.pH≤7.05 | 1.7 (39) | 1.5 (144) | 1.09 (0.77;1.56) | 1.19 (0.82;1.73) |
| Transfer to NCU | 6.3 (147) | 5.5 (519) | 1.15 (0.95;1.39) | 1.22 (0.99;1.50) |
| Early discharge | 33.0 (770) | 30.2 (2841) | 1.14 (1.03;1.25) | 1.03 (0.91;1.17) |

*Adjusted for maternal age, parity, maternal pre-pregnancy BMI, birth weight, smoking habits, need for interpreter, maternity unit, and birth year. We also controlled for pre-pregnancy risks which included: previous IUGR, caesarean sections, and preterm births., and for complications during pregnancy which included: malformations; alcohol or drug abuse; IVF; primiparous<20; preeclampsia; hypertension; diabetes; premature contractions < 37 weeks of gestation; vaginal bleeding <37 weeks of gestation; placental abnormalities; uterine abnormalities, and blood type incompatibilities (Rh, ABO, platelets, hydrops foetalis, and other kinds of blood type incompatibilities).
