# Supplementary material for: Labour outcomes in caseload midwifery and standard care: a register-based cohort study
Source: BMC Pregnancy Childbirth. 2018 Dec 6;18:481. doi: 10.1186/s12884-018-2090-9 (PMC6282374; doi:10.1186/s12884-018-2090-9)
Supplement: Supplementary file 7 — Table S7. Labour outcomes in caseload midwifery and standard care - Outcomes in the present study and findings from nine different international studies. (DOCX 37 kb) [file 12884_2018_2090_MOESM7_ESM.docx]

Table S7) Labour outcomes in caseload midwifery and standard care - Outcomes in the present study and findings from nine different international studies

|  | Present study  2016  Denmark  Cohort  All p all risk All p. low^1^ risks  n=13115 n=6506 | | | | |  | M@ngo 2013  Australia  RCT  All p all risks  n= 1748 | |  | Cosmos  2012  Australia  RCT  All p low risks  n=2314 | |  | Beckmann  2011  Australia  Cohort  All p mixed risks^2^  n=15425 | |  | Tracy  2014  Australia  Cross sect.  All p. low risks  n=1379 | |  | Gottvall  2011  Sweden  Cohort  low risks^3^ primi  n=11937 | |  |
| --- | --- | --- | --- | --- | --- | --- | --- | --- | --- | --- | --- | --- | --- | --- | --- | --- | --- | --- | --- | --- | --- |
|  | CM | SC |  | CM | SC |  | MGP | SC |  | CM | SC |  | MGP | SC |  | MGP | SC |  | MBC | SC |  |
|  | % | % |  | % | % |  | % | % |  | % | % |  | % | % |  | % | % |  | % | % |  |
| Caesarean Sections |  |  |  |  |  |  |  |  |  |  |  |  |  |  |  |  |  |  |  |  |  |
| Before labour | 8.4 | 7.6 |  | 3.0 | 3.2 |  | 8^4^ | 11^4^ |  | 3,1 | 3,5 |  | CS excluded | |  | 5.7 | 17.9 |  | 3.6 | 7.3 |  |
| With labour | 16.5 | 14.5 |  | 9.4 | 8.4 |  | 13 | 13 |  | 16.3 | 21.4 |  | 14.7 | 16.6 |  | 11.5 | 15.1 |  | 15.1 | 18.2 |  |
| Induction | 26.0 | 25.8 |  | 12.4 | 11.7 |  | 24 | 28 |  |  |  |  | 14.7 | 16.0 |  | 19.1 | 27.7 |  | 16.6 | 19.4 |  |
| augmentation (syntocinon) | 22.1 | 21.8 |  | 20.9 | 20.5 |  | 25 | 21 |  |  |  |  | 35.6 | 38.7 |  | 35.5^5^ | 35.2^5^ |  | 31.7^6^ | 32.3^6^ |  |
| epidural | 24.4 | 26.2 |  | 20.0 | 21.4 |  | 36 | 35 |  | 30.5 | 34.6 |  | 28.4 | 33.5 |  | 27.8 | 34.4 |  | 37.6 | 53.8 |  |
| Instrumental birth | 5.8 | 6.5 |  | 6.7 | 6.8 |  | 20 | 19 |  | 17.7 | 19.4 |  | 14.7 | 14.3 |  | 14.5 | 17.9 |  | 15.4 | 16.8 |  |
| Low Apgar^7^ score at 5 min | 2.0 | 1.3 |  | 1.2 | 1.0 |  | 4 | 4 |  | 1.4 | 1.9 |  |  |  |  | 2.1 | 3.4 |  | 0.7 | 1.3 |  |
| Admitted to NCU | 6.2 | 5.5 |  | 4.6 | 3.4 |  | 11 | 12 |  | 4.0 | 6.4 |  |  |  |  | 8.4 | 15 |  |  |  |  |
| Premature birth before 37 weeks | 6.9 | 6.6 |  | 5.9 | 4.4 |  | 4 | 6 |  | 3.8 | 4.1 |  |  |  |  |  |  |  |  |  |  |
| intact perineum | 65.8 | 59.8 |  | 64.6 | 57.3 |  | 13 | 12 |  |  |  |  |  |  |  |  |  |  |  |  |  |
| laceration 1 and 2 | 32.1 | 37.7 |  | 33.1 | 40.1 |  | 46 | 41 |  |  |  |  |  |  |  |  |  |  |  |  |  |
| laceration 3 and 4 | 2.3 | 2.9 |  | 2.2 | 2.9 |  | 4 | 3 |  | 5.0 | 5.3 |  |  |  |  |  |  |  | 6 | 7.3 |  |
| episiotomy | Overall episiotomy rate 4% | | | | |  | 19 | 22 |  |  |  |  |  |  |  |  |  |  | 5.1 | 8.8 |  |

Table 7 continued

|  | Begley  2011  Ireland  RCT  All p low risk  n=1653 | |  | Page  2001  UK  Cohort  All p all risk  n=1403 | |  | Eide  2009  Norway  Cohort  Low risk primi  n=453 | |  | Wernham  2016  New Zealand  Cohort  All p all risks  n=244047 | |  |
| --- | --- | --- | --- | --- | --- | --- | --- | --- | --- | --- | --- | --- |
|  | MLU | CLU |  | 1-1 | CC |  | MLW | CDW |  | Mid-led | Med-led |  |
|  | % | % |  | % | % |  | % | % |  |  |  |  |
| Caesarean Sections |  |  |  |  |  |  |  |  |  |  |  |  |
| Before labour |  |  |  | All CS  19 18 | |  |  |  |  |  |  |  |
| With labour | 14.8 | 15.2 |  |  |  |  | 6.3 | 7 |  |  |  |  |
| Induction | 22.5 | 25 |  | 17 | 24 |  |  |  |  |  |  |  |
| augmentation (syntocinon) |  |  |  | 24 | 33 |  |  |  |  |  |  |  |
| epidural | 18.3 | 24.3 |  | 52 | 67 |  | 24.2 | 62.7 |  |  |  |  |
| Instrumental birth | 12.6 | 14.3 |  | 14 | 19 |  | 11.5 | 12 |  |  |  |  |
| Low Apgar^7^ scores at 5 min | 0.9 | 1.6 |  | 0 | 1 |  | no | diff |  | 0.50 | 0.24 |  |
| Admitted to NCU | 11.6 | 10.9 |  | 5 | 5 |  | no | diff |  |  |  |  |
| befor 37 weeks |  |  |  |  |  |  |  |  |  |  |  |  |
| intact perineum | 38.2 | 40.8 |  | 34 | 26 |  |  |  |  |  |  |  |
| laceration 1 and 2 |  |  |  |  |  |  |  |  |  |  |  |  |
| laceration 3 and 4 |  |  |  |  |  |  | 14 | 11 |  |  |  |  |
| episiotomy | 11.4 | 12.3 |  | 19 | 30 |  |  |  |  |  |  |  |

| ^1)^ Definition of Low risk: A singleton pregnancy considered low risk at recruitment: no Pre-pregnancy risks including: former IUGR, caesarean sections and preterm birth. No complications during pregnancy including: malformations, abuse of alcohol or drugs, IVF, primiparous<20, preeclampsia, hypertension, diabetes, premature contractions< 37 weeks of gestation, vaginal bleeding <37 weeks of gestation, placenta abnormalities, uterus abnormalities and immunisation. pre-pregnancy bmi<17 or bmi>30, no more than three abortions or miscarriages, speaking and reading Danish (women needing interpreter excluded) |
| --- |
| 2)Beckman: Women self-selected into MGP (four midwives). Low risk from 2006-2009, and all risks from 2009-2010. Inclusion criteria for standard care : singleton, planning vg birth, delivered after 37 weeks. Excluded infants with morphological or chromosomal abnormality. |
| 3) In “Gottwall” all results are reported in primiparous and multiparous, here primiparous low risk are chosen |
| 4) M@ngo: From start women who wanted Elective Caesarean were excluded |
| 5) Tracy: Only primipara |
| 6) Gottwall: Dystocia |
| 7) Definition of low Apgar score at five minutes.  M@ngo: Apgar ≤7  Cosmos: Apgar < 7  Tracy: Apgar <7  Gottwall: Apgar <7  Begley Apgar < 8  Page: Apgar<7  Eide: Apgar<7  Wernham: Apgar<7 |

*A*bbreviations:

All p all risks: Primiparous and multiparous at all risks included

All p Low risk: Primiparous and multiparous at low risks included

All p mixed risks: Primiparous and multiparous at low risks and later on all risks included (se note 2)

Low risk primi: Primiparous at low risk

CM: Caseload Midwifery

SC: Standard Care

MGP: Midwifery Group Practice

MBC: Modified Birth Center Care

SCG: Standard Care

MLU: Midwife-led unit

CLU: Consultant-led unit

1-1: One-to-One Midwifery practice

CC: Conventional care

MLW: Midwife-led ward

CDW: Conventional delivery ward

NCU: Neonatal care unit

Mid-led: Midwife-Led

Med-led: Medical-Led
